# Transcriptomic Analysis Reveals Adaptive Evolution and Conservation Implications for the Endangered *Magnolia lotungensis*

**DOI:** 10.3390/genes15060787

**Published:** 2024-06-14

**Authors:** Chenyu Shi, Yanjun Xie, Delong Guan, Guole Qin

**Affiliations:** 1 Guangxi Key Laboratory of Sericulture Ecology and Applied Intelligent Technology, Hechi University, Hechi 546300, China; shichenyu521@163.com (C.S.); xyanjun139@163.com (Y.X.); 2 Guangxi Collaborative Innovation Center of Modern Sericulture and Silk, Hechi University, Hechi 546300, China; 3 School of Chemistry and Bioengineering, Hechi University, Hechi 546300, China

**Keywords:** *Magnolia lotungensis*, RNA-seq, genetic adaptability, species-specific genes, codon usage bias, conservation genetics

## Abstract

*Magnolia lotungensis* is an extremely endangered endemic tree in China. To elucidate the genetic basis of *M. lotungensis*, we performed a comprehensive transcriptome analysis using a sample integrating the plant’s bark, leaves, and flowers. De novo transcriptome assembly yielded 177,046 transcripts and 42,518 coding sequences. Notably, we identified 796 species-specific genes enriched in organelle gene regulation and defense responses. A codon usage bias analysis revealed that mutation bias appears to be the primary driver of selection in shaping the species’ genetic architecture. An evolutionary analysis based on dN/dS values of paralogous and orthologous gene pairs indicated a predominance of purifying selection, suggesting strong evolutionary constraints on most genes. A comparative transcriptomic analysis with *Magnolia sinica* identified approximately 1000 ultra-conserved genes, enriched in essential cellular processes such as transcriptional regulation, protein synthesis, and genome stability. Interestingly, only a limited number of 511 rapidly evolving genes under positive selection were detected compared to *M. sinica* and *Magnolia kuangsiensis*. These genes were enriched in metabolic processes associated with adaptation to specific environments, potentially limiting the species’ ability to expand its range. Our findings contribute to understanding the genetic architecture of *M. lotungensis* and suggest that an insufficient number of adaptive genes contribute to its endangered status.

## 1. Introduction

*M. lotungensis*, a rare and endemic tree species belonging to the Magnoliaceae family, is a unique botanical treasure found exclusively in China [1,2]. As an evergreen tree, *M. lotungensis* can reach impressive heights of 20–30 m, boasting leathery leaves, white flowers, and elliptical aggregate fruits. Its flowering period spans from April to May, while the fruiting season occurs between August and September. The distribution of this species is limited to the southern provinces of China, including Hainan, Guangdong, and Guangxi, where it thrives in the fertile broadleaf forests at elevations ranging from 700 to 1400 m [3,4,5].

Despite its ecological and evolutionary significance, *M. lotungensis* faces severe threats to its survival [6,7]. The species’ restricted distribution, habitat fragmentation, and anthropogenic disturbances, coupled with its low reproductive rate and long growth cycle, have led to a drastic decline in population size. Current estimates suggest that fewer than 2500 mature individual plants remain in the wild, prompting the International Union for Conservation of Nature (IUCN) to assess *M. lotungensis* as an endangered species and the Chinese government to designate it as a national second-class protected plant [6,7,8,9,10].

Unraveling the genetic blueprint of *M. lotungensis*, encapsulated within its transcriptome, holds the key to understanding its adaptability, resilience, and susceptibility to extinction pressures [4,7]. However, our current understanding of *M. lotungensis* at the genetic level is severely limited. A thorough investigation of publicly available databases reveals a paucity of accessible genetic sequences for this species. The only available resources include the chloroplast organelle genome (Genbank ID: NC_062929, 160,046 bp), which serves as a non-nuclear molecular marker [1], and a mere two mRNA coding sequence (CDS) entries for nuclear genes (WRKY33: Genbank ID MG932087.1 and HSP90: Genbank ID MG932086.1). Concerning sequencing data, only four low-coverage DNA sequencing datasets (NCBI SRA database ID: SRR12807275, SRR23908992, SRR23908994, and SRR23908995) are available [11,12]. These datasets, comprising 12.3 G~19.3 G bases, yield an estimated coverage of merely 10× when compared to the typical genome size of other known Magnolia species, which generally fall around 2~3 Gb [12,13]. Such low coverage is grossly insufficient for de novo assembling or aligning the data to other species’ genomes to obtain accurate species-specific genes.

This study presents an overview of the *M. lotungensis* transcriptome, derived from a comprehensive sample integrating the plant’s bark, leaves, and flowers. While the transcriptome may not represent the zenith of genomic exploration, falling short of the exhaustive nature of whole-genome sequencing, it nevertheless provides an economically viable and pragmatic approach that resonates with the pressing conservation imperatives. The de novo assembly of the transcriptome, notwithstanding its inherent constraints, establishes a robust foundation upon which future genome-scale investigations can be built [14,15,16]. By delving into the structural and evolutionary patterns embedded within these genes, we can unravel the intricate interplay between mutation bias and natural selection constraints. Examining codon usage bias allows us to discern the distinct roles played by these forces in shaping the genetic architecture of the species [17,18]. Moreover, by scrutinizing the dN/dS substitution rates among paralogous genes within *M. lotungensis*, we can shed light on the evolutionary trajectory of gene families, gaining insights into the molecular mechanisms that underpin the species’ adaptations and vulnerabilities [19,20,21].

Herein, the transcriptomic analysis of *M. lotungensis* presented in this study not only enriches our understanding of this endangered species at the genetic level but also provides actionable insights crucial for its conservation. Through this multifaceted approach, we can begin to piece together the puzzle of *M. lotungensis*’ population decline and endangerment, identifying the underlying genetic factors that have rendered it susceptible to the challenges it faces. The identification of key genes and the elucidation of genetic patterns within the transcriptome offer a multifaceted perspective on the evolutionary history and current plight of *M. lotungensis*. By unraveling the complex interplay between genetic structure, sequence substitutions, and evolutionary pressures, we can paint a more comprehensive picture of the species’ plight and inform targeted conservation strategies that aim to mitigate the threats it encounters. As a clarion call for integrating advanced molecular techniques into conservation biology, this research advocates for a proactive, informed approach to safeguarding this endangered plant. 

## 2. Materials and Methods

### 2.1. Sample Collection and RNA Extraction

Samples of *M. lotungensis* were collected from the Mulun Nature Reserve (107.98° E, 25.06° N) in Hechi, Guangxi Province, China. Bark, leaf, and floral tissues were collected from three mature *M. lotungensis* individual trees (>5 years old) with similar rearing conditions and immediately flash-frozen in liquid nitrogen in 2019. About 200 g of tissues were collected, and the samples were then transported to Hechi University for RNA extraction, preserved in a −80 °C refrigerator. 

Total RNA was isolated using a Qiagen RNeasy Plant Mini Kit (Qiagen, Hilden, Germany) following the manufacturer’s protocol, with ~5 g plant samples. The quality and quantity of the extracted RNA were assessed using a NanoDrop 2000 spectrophotometer (Thermo Fisher Scientific, Waltham, MA, USA) and an Agilent 2100 Bioanalyzer (Agilent Technologies, Santa Clara, CA, USA). Only RNA samples with an RNA Integrity Number (RIN) ≥ 8.0 and a 260/280 ratio between 1.8 and 2.0 were used for subsequent library preparation and sequencing.

### 2.2. Library Preparation and Sequencing 

The qualified RNA samples were sent to Personalbio Biotechnology Co., Ltd. (Shanghai, China) for RNA-seq library construction and sequencing. Approximately 50 μg of total RNA was used as input material for library construction. The library was prepared using the NEBNext Ultra RNA Library Prep Kit for Illumina (New England Biolabs, Ipswich, MA, USA) following the manufacturer’s instructions. In brief, mRNA was enriched from total RNA using oligo (dT) magnetic beads and then fragmented into short pieces using divalent cations under elevated temperature. The fragmented mRNA was reverse-transcribed into first-strand cDNA using random hexamer primers and M-MuLV Reverse Transcriptase (RNase H-). Second-strand cDNA synthesis was subsequently performed using DNA Polymerase I and RNase H. The double-stranded cDNA fragments were end-repaired, A-tailed, and ligated with sequencing adapters. The ligated products were size-selected using AMPure XP beads (Beckman Coulter, Brea, CA, USA) and PCR-amplified to generate the final cDNA libraries. The quality of the library was assessed using the Agilent 2100 Bioanalyzer, which determined the total library concentration and effective library concentration. The highly qualified library was then sequenced on the Illumina HiSeq 2500 platform (Illumina, San Diego, CA, USA) with a paired-end 150 bp (PE150) sequencing strategy. A sufficient data volume of 2.96 GB was obtained.

### 2.3. Data Preprocessing and Transcriptomic Analysis

The raw sequencing data were subjected to quality control using the FastQC software (version 0.11.9) [22]. Reads containing adapters, shorter than 50 bp, or with an average quality score below Q20 were removed using Trimmomatic (version 0.39) [23]. The remaining clean reads of 9.70 Gb were used for subsequent transcriptome assembly and analysis. The clean reads were de novo assembled into transcripts using the Trinity software (version 2.11.0) [24] with default parameters. 

To obtain non-redundant unigene sequences, the assembled transcripts were further processed using the extract_longest_isoforms_from_TrinityFasta.pl within Trinity. The unigenes were then subjected to coding sequence (CDS) and open reading frame (ORF) prediction using TransDecoder (version 5.5.0) [25]. The predicted CDSs were functionally annotated using the BLAST algorithm (version 2.10.1) [26] against various databases, including the NCBI non-redundant protein (NR) database, Gene Ontology (GO) database, and Interproscan database with the SMART, Pfam, SUPERFAMILY, CDD, and the default InterPro applications being used [27]. The E-value threshold was set to 1 × 10^−5^ for all BLAST searches. The top hit for each CDS was retained for functional annotation. 

### 2.4. Bioinformatic Analysis

To investigate the codon usage bias and evolutionary patterns of the *M. lotungensis* transcriptome, the predicted CDSs were analyzed using DnaSP (version 6.12.03) [28]. The effective number of codons (ENC) and the relative synonymous codon usage (RSCU) were calculated to assess codon usage bias. To examine the selective pressures acting on the *M. lotungensis* transcriptome, the rates of synonymous (dS) and non-synonymous (dN) substitutions were estimated using the codeML (version 4.10.6) [29] implemented in the WGD pipeline (version 1.1.2) [30]. Genomes and CDS files of *M. kwangsiensis* (GCA_037074715.1) and *M. sinica* (GCF_029962835.1) were downloaded from the NCBI Assembly database, and their identified protein sequences were used for a homolog gene search using Orthofinder (version 2.5.5) [31]. The dN/dS ratio was calculated across CDS gene pairs, with values greater than 1 indicating positive selection, values equal to 1 suggesting neutral evolution, and values less than 1 implying purifying selection. The results of the codon usage bias and dN/dS analyses were visualized using the ggplot2 package (version 3.3.3) [32] in R (version 4.0.4) [33]. Other figures were visualized using the OmicStudio tools at https://www.omicstudio.cn/tool, accessed on 3 May 2024.

## 3. Results

### 3.1. De Novo Assembly of the M. lotungensis Transcriptome

The comprehensive transcriptome of *M. lotungensis* was reconstructed using the Trinity software, employing a de novo assembly approach. The assembly yielded a total of 177,046 transcripts and 138,343 unigenes (Supplementary File S1). The longest sequence in both the transcript and unigene sets was 9478 base pairs (bp), while the shortest transcript and unigene were 187 bp and 201 bp, respectively. The total size of the assembled transcriptome was 86,522,543 bp, with the unigene set accounting for 58,499,986 bp. The GC content of the transcripts and unigenes was 50.09% and 50.73%, respectively, indicating a balanced nucleotide composition.

The assembly quality was assessed using various metrics, including N50 and L50 values. The N50 value, which represents the length of the shortest sequence among the longest sequences that collectively cover 50% of the total assembly size, was 590 bp for transcripts and 445 bp for unigenes. The corresponding L50 values, which denote the number of sequences with lengths equal to or greater than the N50 value, were 37,818 for transcripts and 32,890 for unigenes. Similarly, the N90 and L90 values were calculated, with N90 being 240 bp and 229 bp for transcripts and unigenes, respectively, and L90 values of 137,677 and 111,122, respectively (Supplementary File S1).

To identify potential protein-coding sequences (CDSs) within the assembled unigenes, TransDecoder was employed. A total of 42,518 CDSs were extracted from the unigene set, with a cumulative length of 25,855,698 bp. The longest CDS was 6069 bp, while the shortest was 255 bp. The GC content of the CDS set was slightly higher than that of the transcripts and unigenes, at 51.83%. The N50 and L50 values for the CDS set were 660 bp and 11,460, respectively, indicating higher contiguity compared to the transcript and unigene sets. The N90 and L90 values for the CDS set were 339 bp and 34,301, respectively (Supplementary File S1).

Of the 42,518 CDSs predicted using TransDecoder, a total of 37,271 (87.7%) were successfully annotated by at least one of the databases employed in this study (Supplementary File S2). The NCBI non-redundant protein (nr) database provided the highest number of annotations, with 36,072 (84.8%) CDSs finding significant hits. This extensive coverage demonstrates the effectiveness of the NR database in capturing a broad range of protein functions across diverse species. The Pfam database, which contains curated protein families and domains, annotated 26,731 (62.9%) CDSs, offering valuable information on conserved functional domains within the *M. lotungensis* transcriptome. The Gene Ontology (GO) and InterPro (IPR) databases, which provide a standardized vocabulary for describing gene products and their attributes, annotated an equal number of 29,873 (70.3%) CDSs. The Superfamily database, which provides structural and functional annotations for protein domains and families, annotated 22,372 (52.6%) CDSs. This database is particularly useful for identifying distant evolutionary relationships among proteins and inferring potential functions based on structural similarities. The Conserved Domain Database (CDD) and SMART database, which focus on conserved protein domains, annotated 7284 (17.1%) and 6929 (16.3%) CDSs, respectively.

**Figure 1:**
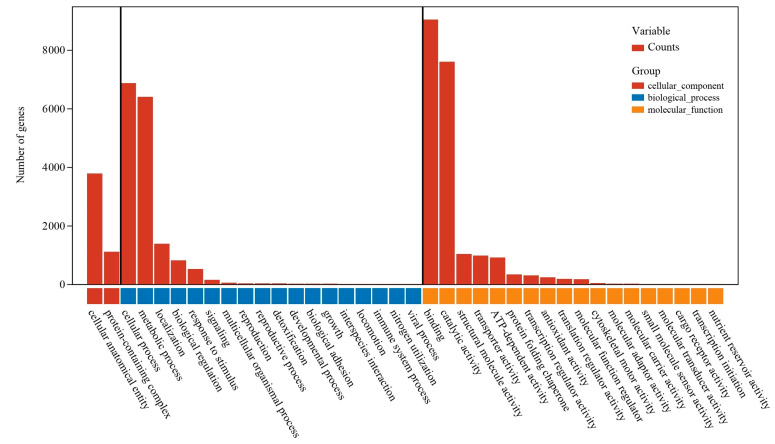
Gene Ontology (GO) annotation and classification of the *M. lotungensis* transcriptome.

To gain insights into the overall functional roles of the identified genes in the *M. lotungensis* transcriptome, we performed Gene Ontology (GO) annotation using the GO database (Figure 1). Within the cellular component category, “cellular anatomical entity” was the most abundant term, with 3782 genes assigned, followed by “protein-containing complex” with 1104 genes. These results suggest that a significant portion of the transcriptome is dedicated to the structural and functional organization of cells. The biological process category encompassed a wide range of functions, with the “cellular process” (6865 genes) and “metabolic process” (6391 genes) being the most heavily represented. These findings highlight the importance of basic cellular processes and metabolic pathways in the overall functioning of *M. lotungensis*. Other notable biological processes included “localization” (1382 genes), “biological regulation” (807 genes), and “response to stimulus” (516 genes), indicating the plant’s ability to adapt and respond to various environmental cues. Interestingly, a small number of genes were associated with specialized processes such as “detoxification” (23 genes), “interspecies interaction” (7 genes), and “nitrogen utilization” (1 gene), suggesting potential adaptations to specific ecological niches. The molecular function category was dominated by “binding” (9032 genes) and “catalytic activity” (7599 genes), underlining the importance of molecular interactions and enzymatic reactions in the plant’s cellular machinery. “Structural molecule activity” (1034 genes) and “transporter activity” (974 genes) were also well represented, reflecting the need for maintaining cellular structure and facilitating the movement of molecules within and between cells. Additionally, a considerable number of genes were involved in “ATP-dependent activity” (909 genes) and “protein folding chaperone” functions (329 genes), emphasizing the role of energy-driven processes and protein quality control in the plant’s physiology. The GO annotation results provide a comprehensive overview of the diverse functions and processes represented in the *M. lotungensis* transcriptome. The high proportion of genes involved in fundamental cellular processes, metabolism, and molecular interactions underscores the importance of these pathways in the plant’s survival and adaptation. Furthermore, the presence of genes associated with specific ecological adaptations and stress responses offers valuable insights into the genetic mechanisms underlying *M. lotungensis*’ resilience and vulnerability. These findings lay the groundwork for further investigations into the molecular basis of *M. lotungensis*’ unique biology and conservation challenges. 

**Figure 2:**
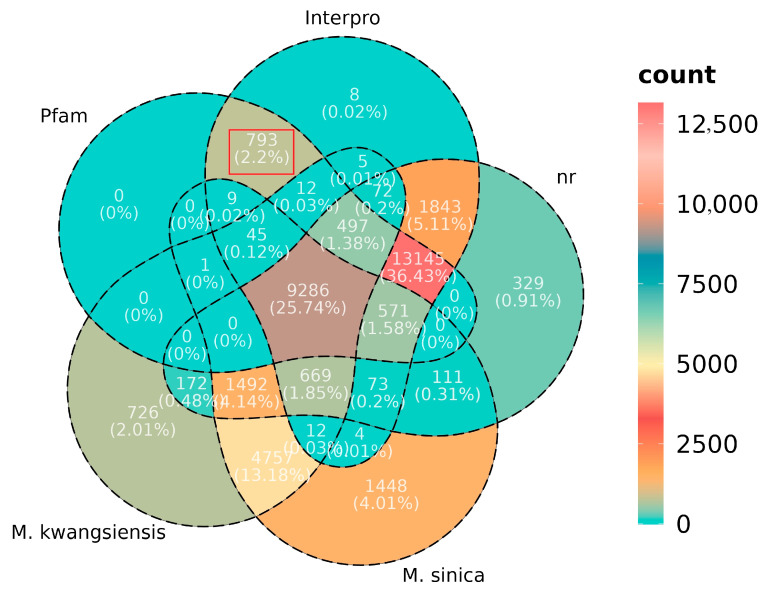
Venn diagram depicting the overlap and uniqueness of Pfam and InterPro domain annotations among *M. lotungensis*. The red box represents the 793 elements unique to *M. lotungensis* correspond to the novel genes identified in its transcriptome, which are absent in the NR database and the genomes of its closely related species.

In diving deep into the functional annotations of the *M. lotungensis* transcriptome, we uncovered a fascinating set of 793 novel gene orthologue groups that exhibited significant hits in the Pfam and InterPro databases (Figure 2, Supplementary File S3). Intriguingly, these genes were not annotated in the NR database and were absent from the high-quality genomes of *M. kwangsiensis* and *M. sinica*, two closely related species. This discovery suggests that these newly identified genes may have undergone independent evolution in *M. lotungensis*, potentially contributing to its unique adaptations and ecological niche. These genes represent a valuable resource for exploring the distinctive genetic repertoire of *M. lotungensis*, offering insights into the molecular mechanisms that have shaped its evolutionary trajectory and enabled its survival in specific habitats. 

**Figure 3:**
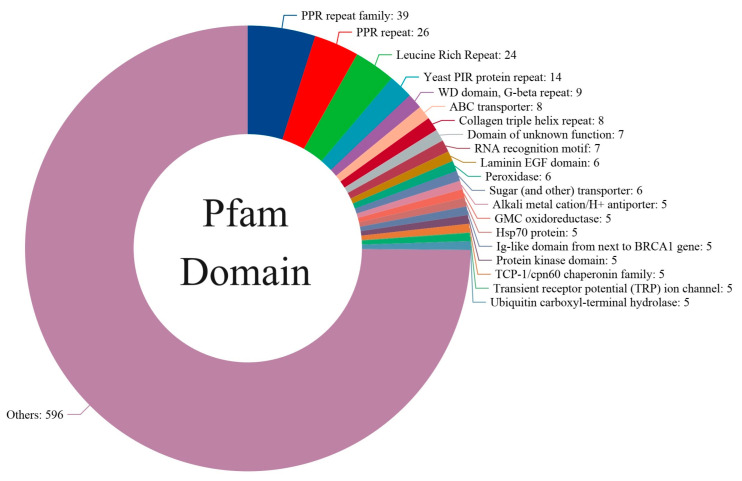
Distribution of Pfam domains among the 793 novel genes identified in the *M. lotungensis* transcriptome.

To gain insights into the functional roles of the 796 novel genes within the 793 orthogroups, we analyzed the distribution of Pfam domains within this set (Figure 3, Supplementary File S3). Overall, the high representation of PPR proteins, LRR domains, and other functional categories suggests that these novel genes may be involved in critical processes such as organelle gene regulation, defense responses, cellular communication, and transport. The most abundant domain was the PPR (pentatricopeptide repeat) family, with 39 genes containing this domain. PPR proteins are known to play crucial roles in organelle gene expression and RNA editing, suggesting that these novel genes may be involved in the regulation of organellar functions in *M. lotungensis*. Additionally, 26 genes were found to harbor the PPR repeat domain, further emphasizing the prevalence and importance of PPR proteins in this species.

Another prominent domain among the novel genes was the leucine-rich repeat (LRR) domain, present in 24 genes. LRR domains are known to mediate protein–protein interactions and are commonly found in plant resistance genes, implicating these novel genes in potential defense mechanisms against pathogens or environmental stressors. The yeast PIR (protein with internal repeats) protein repeat and WD domain (G-β repeat) were also well represented, with 14 and 9 genes, respectively. These domains are often associated with cell wall integrity, signal transduction, and protein–protein interactions, indicating that these novel genes may contribute to the overall cellular communication and structural stability of *M. lotungensis*.

Interestingly, eight novel genes were annotated with ABC transporter domains, suggesting their involvement in the transport of various substrates across cellular membranes. These transporters play essential roles in nutrient uptake, xenobiotic detoxification, and responses to environmental stimuli, highlighting the potential adaptive significance of these genes in *M. lotungensis*. Another eight genes contained the collagen triple helix repeat domain, which is associated with extracellular matrix structure and cell adhesion, hinting at their possible roles in maintaining tissue integrity and cell–cell interactions.

Seven novel genes were classified as having domains of unknown function (DUF), presenting an opportunity for the further characterization and discovery of novel gene functions specific to *M. lotungensis*. Other notable domains identified among the novel genes include the RNA recognition motif (seven genes), laminin EGF domain (six genes), peroxidase domain (six genes), and sugar transporter domain (six genes). These domains are associated with a wide range of biological processes, such as RNA binding, extracellular matrix interactions, oxidative stress response, and carbohydrate transport, respectively.

### 3.2. Codon Usage Bias

**Figure 4:**
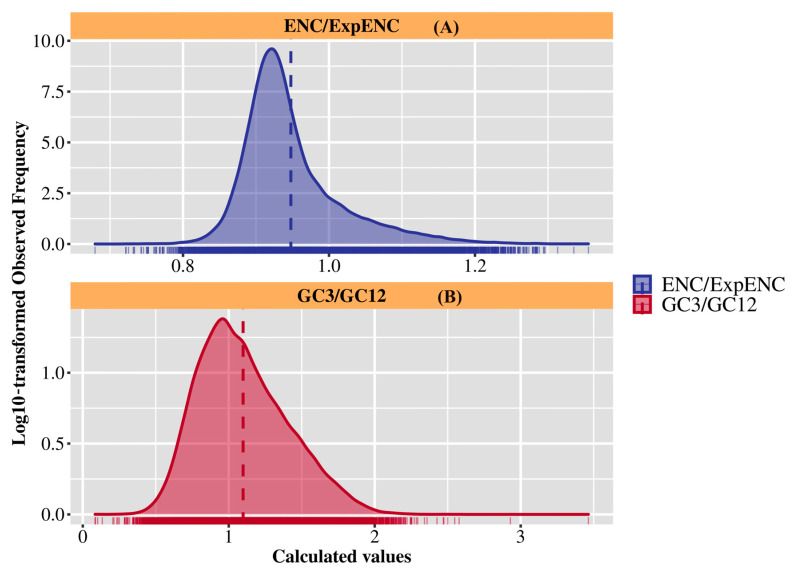
Codon usage bias and GC content analysis in the *M. lotungensis* transcriptome. (**A**) ENC (effective number of codons) plotted against the expected ENC based on GC3 content. The solid curve represents the expected ENC under the assumption of no selection. Genes falling below the curve are considered to exhibit codon usage bias. (**B**) Distribution of GC3/GC ratio, with GC3 representing the GC content at the third codon position and GC representing the overall GC content of the coding sequence.

To investigate the factors influencing codon usage bias in the *M. lotungensis* transcriptome, we analyzed the relationship between the effective number of codons (ENC) and the expected ENC (ExpENC), as well as the ratio of GC content at the third codon position (GC3) to that of the first two codon positions (GC12) (Figure 4, Supplementary File S4). The ENC is a measure of codon usage bias, with lower values indicating stronger bias. ExpENC is the expected ENC under the assumption of no selection, calculated based on the GC content of the coding sequences.

The ENC/ExpENC plot (Figure 4A) reveals a distinct pattern, with most genes exhibiting ENC values lower than the expected values under neutral evolution. This deviation suggests that the observed codon usage bias in the *M. lotungensis* transcriptome is stronger than would be expected in the absence of selection, indicating the influence of selective pressures on codon usage. Notably, the distribution of ENC/ExpENC values forms a bimodal pattern, with peaks around 0.5 and 1.0. This negative binomial distribution suggests the presence of two groups of genes in the transcriptome, one under stronger selective pressure and the other under weaker selective pressure.

Figure 4B presents the distribution of GC3/GC12 values, which provides insights into the relative GC content at different codon positions. The majority of genes in the *M. lotungensis* transcriptome have GC3/GC12 ratios greater than 1, indicating a higher GC content at the third codon position compared to the first two positions. The higher GC content at the third codon position may be attributed to mutational biases or selective pressures acting on synonymous codon sites. Interestingly, the GC3/GC12 distribution also exhibits a bimodal pattern, with peaks around 1.0 and 2.0. This negative binomial mirrors the pattern observed in the ENC/ExpENC plot, further supporting the presence of two distinct gene groups with different codon usage characteristics. The genes with higher GC3/GC12 ratios may be subject to stronger selective pressures favoring GC-ending codons, while those with lower ratios may experience weaker selective pressures or different mutational biases (Figure 4).

Combining these two parts, our analysis of codon usage bias and GC content in the *M. lotungensis* transcriptome reveals the presence of selective pressures and mutational biases shaping the codon usage patterns of this endangered species. The bimodal distributions of both ENC/ExpENC and GC3/GC12 ratios suggest the existence of two distinct gene groups with different codon usage characteristics, potentially reflecting varying levels of selective pressure and mutational biases. 

Furthermore, we performed K-means clustering on the relative synonymous codon usage (RSCU) values of all identified coding sequences (CDSs) (Supplementary File S3). The RSCU values were scaled by row and clustered by column to normalize the data and emphasize the relative differences in codon usage across the CDSs. The optimal number of clusters (K) was estimated to be four based on the elbow method and silhouette analysis (Supplementary Figure S1).

**Figure 5:**
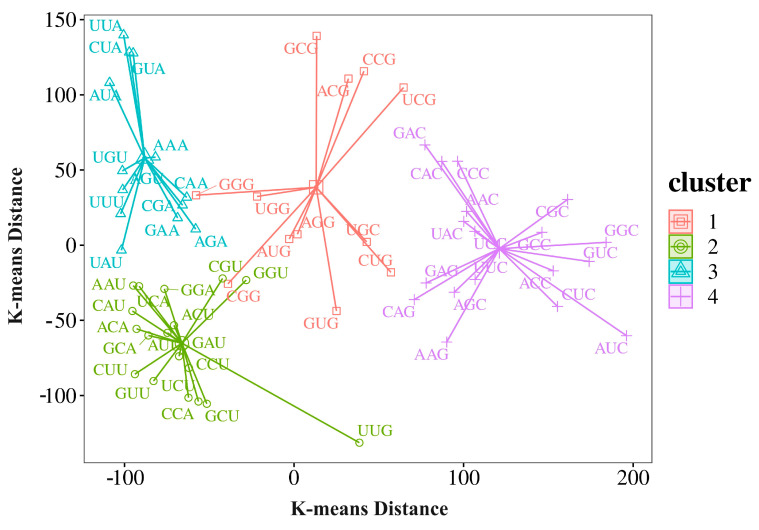
K-means clustering analysis of relative synonymous codon usage (RSCU) values in the *M. lotungensis* transcriptome. RSCU values were scaled by row and clustered by column, revealing four distinct clusters that correspond to the ending nucleotide of each codon. Cluster 1 (orange) represents codons ending with A, cluster 2 (green) represents codons ending with U, cluster 3 (blue) represents codons ending with G, and cluster 4 (magenta) represents codons ending with C.

Intriguingly, the four clusters obtained from the K-means analysis perfectly separated the codons based on their ending nucleotide (Figure 5). This clear segregation of codons based on their third base suggests that the ending nucleotide plays a significant role in shaping the codon usage bias in the *M. lotungensis* transcriptome. This pattern indicates a strong preference for A/U-ending codons over G/C-ending codons in the *M. lotungensis* transcriptome. The prevalence of A/U-ending codons may be attributed to mutational biases, such as a higher mutation rate from G/C to A/U or a more stable DNA conformation associated with A/U-rich sequences. Additionally, selection pressures related to translational efficiency, mRNA stability, or other cellular processes may contribute to the observed codon usage bias.

In summary, our analysis of codon usage bias in the *M. lotungensis* transcriptome reveals the intricate interplay between mutation bias and selection in sculpting the species’ genetic architecture. While both forces contribute to the observed patterns, mutation bias appears to play a more dominant role, as evidenced by the relatively modest deviations of ENC and GC content from neutral expectations. However, strong selection pressures were also detected for a wide array of genes, potentially reflecting the adaptive significance of codon optimization in the face of environmental challenges. This suggests that while mutation bias may be the primary driver of codon usage patterns in *M. lotungensis*, selection has played a crucial role in fine-tuning the genetic composition of certain genes to enhance their functionality and resilience. 

### 3.3. Evolutionary Analysis of Paralogous Gene Pairs in M. lotungensis

**Figure 6:**
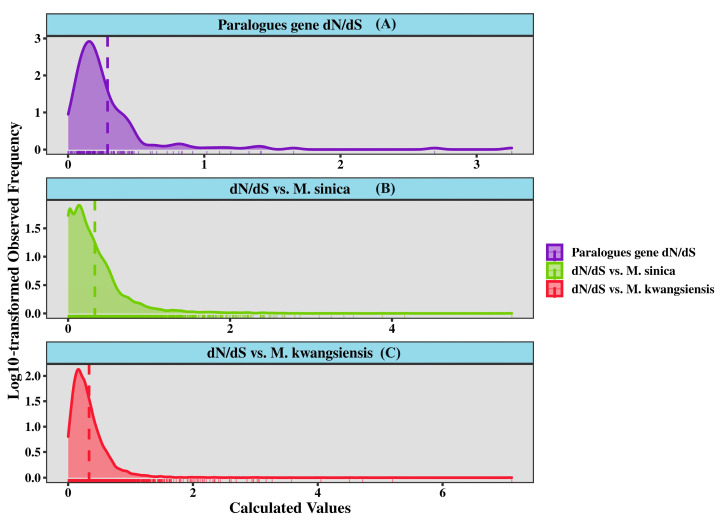
Distribution of evolutionary rates among paralogous gene pairs in the *M. lotungensis* transcriptome. (**A**) The distribution of non-synonymous substitution rates (dN); (**B**) the distribution of synonymous substitution rates (dS); (**C**) the distribution of dN/dS ratios.

To gain insights into the evolutionary dynamics and selective pressures acting on the *M. lotungensis* transcriptome, we investigated the rates of synonymous (dS) and non-synonymous (dN) substitutions between paralogous gene pairs within *M. lotungensis*, as well as orthologous gene pairs between *M. lotungensis* and its closely related species, *M. kwangsiensis* and *M. sinica*. Using the WGD software, we identified gene pairs that met the filtering criteria of alignment coverage greater than 0.6 and identity greater than 0.75 (Figure 6, Supplementary File S5).

Within the *M. lotungensis* transcriptome, we identified 205 paralogous gene pairs, comprising 401 individual genes. The distribution of dN/dS ratios for these paralogous gene pairs exhibited a pronounced peak around 0.1 (Figure 6, purple curve), suggesting that the majority of these genes are under strong purifying selection, which constrains the accumulation of non-synonymous substitutions. This finding highlights the importance of maintaining the functional integrity of these genes and the potential deleterious effects of amino acid changes. Only a small subset of genes experienced positive selection, as indicated by the 18 gene pairs exhibiting dN/dS ratios greater than 1, with 12 of them having functional annotations in the NR database.

The enrichment of positively selected genes in transcriptional regulation, metabolic processes, and stress response suggests their pivotal roles in shaping the evolution and adaptation of *M. lotungensis*. Two genes containing the plant-specific NAC domain, involved in developmental processes and stress responses, were identified. Additionally, two genes were found to be subunits of the Mediator complex, which regulates eukaryotic transcription by facilitating communication between transcription factors and RNA polymerase II. Furthermore, two genes were annotated as NAD-specific glutamate dehydrogenases, catalyzing the reversible oxidative deamination of glutamate. The elevated dN/dS ratios for these genes indicate potential adaptive changes in *M. lotungensis*, influencing its transcriptional regulation and gene expression patterns.

A similar distribution of conserved dN/dS ratios that indicate strong sequence conservation was observed during the comparison of CDS between species. The existence of these highly conserved gene pairs between *M. lotungensis* and *M. sinica* or *M. kwangsiensis* suggests that most genes may also have undergone strong purifying selection, possibly due to their critical functional roles or evolutionary constraints. Alternatively, the low divergence of these genes could be attributed to recent speciation events or ongoing gene flow between the species. A comparative analysis of orthologous gene pairs between *M. lotungensis* and *M. sinica* yielded 7121 gene pairs, while the comparison with *M. kwangsiensis* revealed 7346 gene pairs (Figure 6B,C, Supplementary File S5). The distribution of dN/dS ratios for these orthologous pairs showed a similar pattern to that of the paralogous pairs, with a prominent peak around 0.1, indicating the prevalence of purifying selection in shaping the evolution of these genes across the species. This conservation suggests that these genes may play essential roles in the shared biological processes and adaptations of *M. lotungensis* and its congeners.

Intriguingly, the comparison of orthologous gene pairs between *M. lotungensis* and *M. sinica* yielded 7121 gene pairs and revealed a distinct evolutionary pattern (Figure 6B). In addition to the peak around 0.1, consistent with the findings in other comparisons, a secondary peak was observed, composed of gene pairs with extremely low dN/dS ratios, with a mean value below 0.001. This peak reflects the presence of a substantial number of nearly identical sequences between the two species, indicating a high degree of sequence conservation.

**Figure 7:**
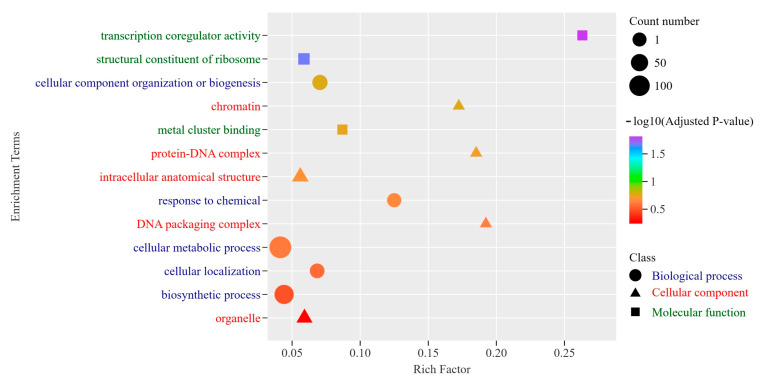
GO enrichment analysis of ultra-conserved genes between *M. lotungensis* and *M. sinica*. The plot displays the enriched GO terms associated with the 951 ultra-conserved genes, categorized into three main classes: biological process (orange), cellular component (green), and molecular function (purple). The *x*-axis represents the rich factor, indicating the proportion of ultra-conserved genes in each GO term relative to the total number of genes in that term. The *y*-axis shows the enriched GO terms, and the size of the dots corresponds to the number of ultra-conserved genes in each term. The color scale on the right represents the adjusted *p*-value (−log10 transformed) for the enrichment significance.

To investigate the functional roles of the ultra-conserved genes shared between *M. lotungensis* and *M. sinica*, we extracted a list of 951 genes with dN/dS ratios below 0.001 and conducted a Gene Ontology (GO) enrichment analysis (Figure 7, Supplementary File S6). A comparative genomic analysis revealed exceptionally high sequence conservation between the two species, with an average alignment coverage of 91.8% and an average identity of 99.34% for these ultra-conserved genes. The GO enrichment results highlighted several key molecular functions, cellular components, and biological processes associated with the ultra-conserved genes (Figure 7). In the molecular function category, the most significantly enriched term was “transcription coregulator activity” (GO:0003712) with an enrichment score of 8.18, suggesting that many of these genes are involved in the regulation of transcription. Other enriched molecular functions included “structural constituent of ribosome” (GO:0003735) and “metal cluster binding” (GO:0051540), indicating the importance of protein synthesis and metal-related processes.

Among the cellular component terms, “organelle” (GO:0043226) and “intracellular anatomical structure” (GO:0005622) were enriched, reflecting the diverse subcellular localization of the ultra-conserved genes. Notably, terms related to DNA packaging and chromatin organization, such as “DNA packaging complex” (GO:0044815), “protein-DNA complex” (GO:0032993), and “chromatin” (GO:0000785), were also significantly enriched, emphasizing the role of these genes in maintaining genome stability and regulation. The biological process category revealed the involvement of ultra-conserved genes in various essential cellular processes. “Cellular component organization or biogenesis” (GO:0071840) and “cellular localization” (GO:0051641) were enriched, indicating the role of these genes in maintaining cellular structure and organization. Additionally, “response to chemical” (GO:0042221) was highly enriched, suggesting that these genes may play a role in the organisms’ ability to adapt to environmental stimuli. Other enriched biological processes included the “biosynthetic process” (GO:0009058) and the “cellular metabolic process” (GO:0044237), highlighting the involvement of ultra-conserved genes in fundamental metabolic pathways.

In addition to the ultra-conserved genes, we also explored the functional roles of the limited number of rapidly evolving genes identified through the comparison of coding sequences (CDSs) between *M. lotungensis* and its closely related species, *M. sinica* or *M. kwangsiensis*. Our analysis revealed that only 511 genes in *M. lotungensis* exhibited dN/dS ratios greater than 1 when compared to either *M. sinica* or *M. kwangsiensis*. The scarcity of these rapidly evolving genes, in stark contrast to the abundance of conserved sequences, underscores the overall lack of sequence diversity in *M. lotungensis*, a finding that aligns with the conclusions drawn in previous sections of our study.

**Figure 8:**
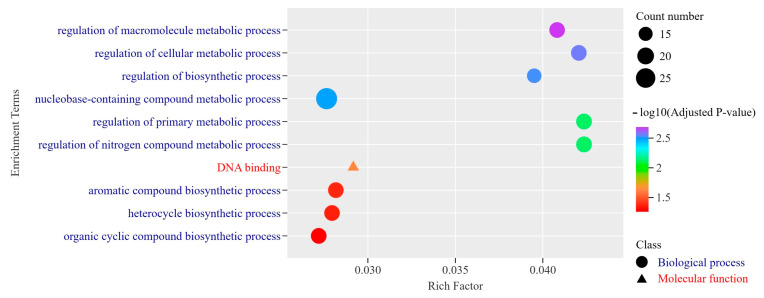
Gene Ontology (GO) enrichment analysis of rapidly evolving genes in *M. lotungensis* compared to *M. sinica* or *M. kwangsiensis*. The bar plot displays the top 10 most significantly enriched GO terms associated with the 511 genes under positive selection (dN/dS > 1) in *M. lotungensis*. The GO terms are categorized into three main classes: biological process (blue), cellular component (green), and molecular function (orange). The *x*-axis represents the enrichment score, calculated as the −log10-transformed adjusted *p*-value for each GO term. The *y*-axis shows the enriched GO terms, with their corresponding GO IDs in parentheses.

To gain insights into the functional categories associated with these rapidly evolving genes, we conducted a Gene Ontology (GO) enrichment analysis (Figure 8, Supplementary File S6). The results of this analysis shed light on the roles of these genes in regulating metabolic processes, biosynthetic pathways, and DNA-related functions in *M. lotungensis* compared to *M. sinica* or *M. kwangsiensis*. The most significantly enriched terms encompassed “regulation of primary metabolic process” (GO:0080090), “regulation of nitrogen compound metabolic process” (GO:0051171), “regulation of cellular metabolic process” (GO:0031323), and “regulation of macromolecule metabolic process” (GO:0060255). These findings suggest that the rapidly evolving genes play a pivotal role in modulating the metabolic activities of the organisms, potentially contributing to their adaptations to distinct environments.

Furthermore, several enriched terms were associated with biosynthetic processes, including “regulation of biosynthetic process” (GO:0009889), “aromatic compound biosynthetic process” (GO:0019438), “heterocycle biosynthetic process” (GO:0018130), and “organic cyclic compound biosynthetic process” (GO:1901362). In the molecular function category, the most significantly enriched term was “DNA binding” (GO:0003677), indicating that a substantial proportion of the rapidly evolving genes encode proteins that interact with DNA and potentially regulate gene expression. These results suggest that the rapidly evolving genes are involved in the synthesis of a diverse array of organic compounds.

## 4. Discussion

The comprehensive transcriptome analysis of *M. lotungensis*, an endangered endemic tree species in China, has provided novel insights into its genetic architecture, codon usage bias, and evolutionary dynamics. The de novo assembly of the transcriptome yielded a robust set of 177,046 transcripts with 42,518 coding sequences (CDSs) identified, of which 37,271 (87.7%) were functionally annotated. Although transcriptome sequencing may not be the most optimal technique for genetic exploration today, given the critically endangered status of *M. lotungensis* and the difficulty in obtaining samples, the quality of the assembled transcriptome is sufficient to provide a comprehensive description of the species’ genetic information, and are comparable to previous studies focused on the *Magnolia* [15,16,34,35,36]. For instance, the de novo transcriptome assembly of *M. wufengensis* and *M. champaca* yielded 59,764 and 47,688 non-redundant unigenes, respectively [15,16]. Before this study, the available sequence information for *M. lotungensis* was extremely limited, with only the chloroplast genome and a few low-coverage NGS sequencing datasets [11]. Therefore, the identification of these lineage-specific genes substantially expands our understanding of the species’ unique genetic composition and provides valuable insights into its distinct evolutionary trajectory. This valuable resource will facilitate future research efforts, such as unraveling the molecular basis of the species’ biological and ecological characteristics, developing primers for population diversity assessment, and designing targeted conservation strategies.

One of the most significant findings of this study is the identification of 796 novel species-specific genes in *M. lotungensis* that are absent in closely related species and common public databases. This discovery suggests the presence of lineage-specific genes that may contribute to the species’ unique ecological adaptations. The enrichment analysis of these novel genes revealed their potential roles in organelle gene regulation, defense responses, cellular communication, and transport, indicating their possible contributions to *M. lotungensis*’ resilience and vulnerability to various abiotic stressors, such as cold stress at higher elevations or soil nutrient limitations. Notably, 39 genes were found to contain the PPR (pentatricopeptide repeat) domain, which is known to play crucial roles in organelle gene expression and RNA editing [37,38]. Additionally, 24 genes harbored the leucine-rich repeat (LRR) domain, commonly found in plant resistance genes and potentially involved in defense mechanisms against pathogens or environmental stressors [39,40,41]. The co-occurrence of PPR and LRR domains in the lineage-specific genes of *M. lotungensis* suggests a possible synergistic effect, where the fine-tuning of organelle gene expression and the reinforcement of defense mechanisms work in concert to enhance the species’ adaptability and resilience. This unique genetic repertoire may have evolved as a result of the selective pressures imposed by the species’ restricted habitat and the need to maintain viable populations in the face of environmental challenges.

To investigate the evolutionary factors influencing gene sequences in *M. lotungensis*, we conducted a codon usage bias analysis. The results revealed that both mutation bias and selection pressure shape the species’ codon usage patterns, with mutation bias playing a more dominant role. The deviation of the effective number of codons (ENC) from the expected values under neutral evolution suggests that the observed codon usage bias is stronger than would be anticipated in the absence of selection. However, the negative binomial distribution of ENC values and the GC3/GC12 ratio indicates that mutation bias is the primary driver of codon usage patterns, with selection pressure having a secondary effect on certain genes. These findings are consistent with previous studies on codon usage in other endangered plant species, such as *Ginkgo biloba* [42] and *Taxus contorta* [43], where sequence diversity tends to decline continuously and is minimally influenced by strong selection pressures. In the case of *M. lotungensis*, the observed codon usage patterns likely reflect the combined effects of random mutations at the genome-wide level, widespread purifying selection, and potentially rare instances of positive selection in a limited number of genes [44,45,46]. 

The evolutionary analysis of paralogous gene pairs within *M. lotungensis* and orthologous gene pairs between *M. lotungensis* and closely related species (*M. sinica* and *M. kwangsiensis*) further supported the notion of limited sequence diversity and the prevalence of purifying selection. The majority of gene pairs exhibited low dN/dS ratios, indicating strong evolutionary constraints and the conservation of gene sequences. The identification of abundant ultra-conserved genes (~1000) between *M. lotungensis* and *M. sinica*, with extremely low sequence divergence, further highlights the high degree of evolutionary conservation among closely related species, which suggests a deficiency in the emergence of novel genes during the processes of species divergence and independent evolution. This lack of genetic innovation may hinder the species’ ability to adapt to changing environmental conditions and contribute to its endangered status [47,48,49]. Our findings emphasize the importance of sequence conservation in the biology of *M. lotungensis* and provide compelling evidence that the paucity of new genes arising from adaptive evolution is a key factor underlying its threatened status. These insights underscore the critical need for conservation efforts focused on maintaining the genetic diversity of *M. lotungensis* and promoting the generation of novel adaptive genes to ensure its long-term survival.

The identification of a small number of rapidly evolving genes further supports our hypothesis that the lack of sufficient novel genes during species divergence and independent evolution is a major contributing factor to the endangered status of *M. lotungensis*. Among the 18 rapidly evolving paralogous gene pairs identified, 12 had annotations in the NR database, with roles related to transcriptional regulation (e.g., NAC domain-containing proteins and Mediator complex subunits), metabolic processes (e.g., NAD-specific glutamate dehydrogenases), and stress responses. The presence of rapidly evolving genes related to transcriptional regulation, such as those containing the NAC domain or encoding subunits of the Mediator complex, highlights the importance of fine-tuning gene expression in response to changing environmental conditions [50]. The NAC domain-containing proteins are plant-specific transcription factors known to regulate various developmental processes and stress responses [51,52]. Similarly, the NAD-specific glutamate dehydrogenases suggest that *M. lotungensis* may have adapted its metabolic pathways to optimize resource utilization and energy production in its specific habitat [53]. Glutamate dehydrogenases catalyze the reversible oxidative deamination of glutamate to α-ketoglutarate and ammonia, playing a crucial role in amino acid metabolism and nitrogen homeostasis [54]. The accelerated evolution of genes related to nitrogen assimilation and utilization in *M. lotungensis* may reflect adaptations to its specific nutritional requirements and soil conditions. However, while these adaptations enhance the species’ fitness in its current habitat, they may also limit its ability to colonize new environments, contributing to its endangered status. The scarcity of novel genes arising from adaptive evolution during species divergence appears to be a critical factor restricting *M. lotungensis*’ potential for widespread dispersal.

The comparative analysis between *M. lotungensis* and closely related species revealed 511 rapidly evolving genes, with functions encompassing various essential cellular processes. The GO enrichment analysis of these rapidly evolving genes revealed their involvement in the regulation of metabolic processes, biosynthetic pathways, and DNA-related functions. The enrichment of rapidly evolving genes in biosynthetic processes, including the “regulation of biosynthetic process”, “aromatic compound biosynthetic process”, “heterocycle biosynthetic process”, and “organic cyclic compound biosynthetic process”, indicates that *M. lotungensis* may have undergone adaptive changes in the production of secondary metabolites. These metabolites, such as aromatic compounds, heterocycles, and organic cyclic compounds, play diverse roles in plant defense, signaling, and environmental interactions [55,56,57,58,59]. The rapid evolution of genes related to their biosynthesis suggests that *M. lotungensis* may have evolved unique chemical defenses or communication strategies to cope with biotic and abiotic stresses in its specific ecological niche.

In summary, the transcriptome analysis of *M. lotungensis* has provided a comprehensive overview of the genetic underpinnings of this endangered species, revealing the complex interplay of evolutionary forces and functional adaptations that have shaped its biology. The scarcity of these positively selected genes, in contrast to the abundance of conserved sequences, underscores the overall lack of sequence diversity in *M. lotungensis*. The ability of these genes to escape the overall trend of sequence conservation and undergo rapid evolution raises questions about their adaptive significance and the selective pressures driving their divergence. This finding is consistent with previous studies on many endangered plants, including other *Magnolia* species [4,12,13,47]. The low genetic diversity may render *M. lotungensis* more vulnerable to environmental changes and biotic stressors, as it may lack the necessary genetic variation to adapt and survive under challenging conditions.

As we continue to unravel the genetic secrets of *M. lotungensis*, it is crucial to integrate these molecular insights with ecological and conservation efforts to ensure the long-term survival of this botanical treasure. The transcriptome resources generated in this study lay the foundation for further investigations into the functional significance of the identified genes, the development of molecular markers for population genetics studies, and the exploration of genetic diversity within and among *M. lotungensis* populations. By bridging the gap between genomic research and on-the-ground conservation initiatives, we can work towards a future where the captivating beauty and ecological importance of *M. lotungensis* are preserved for generations to come.

## Data Availability

The data presented in this study are openly available in Genbank SRA database at https://www.ncbi.nlm.nih.gov/sra/PRJNA1108191, reference number PRJNA1108191.

## Supplementary Materials

The following supporting information can be downloaded at https://www.mdpi.com/article/10.3390/genes15060787/s1: Supplementary File S1: De novo Assembly Metrics and Statistics for *M. lotungensis* Transcriptome; Supplementary File S2: Annotation Summary of Predicted Coding Sequences (CDS) in *M. lotungensis*; Supplementary File S3: Functional Annotations and Novel Gene Orthologue Groups in *M. lotungensis*; Supplementary File S4: Calculated Effective number of codon (ENC), GC Content, and Relative Synonymous Codon Usage (RSCU) values in *M. lotungensis* Transcriptome; Supplementary File S5: Calculated dN/dS values of Paralogous and Orthologous Gene Pairs; Supplementary File S6: Gene Ontology (GO) Enrichment Analysis of Ultra-conserved Genes and rapidly evolved genes in *M. lotungensis*; and Supplementary Figure S1: Elbow Method and Silhouette Analysis for Optimal Cluster Number Estimation.
