# Supplementary figures and images for: Transcriptomic Analysis Reveals Adaptive Evolution and Conservation Implications for the Endangered Magnolia lotungensis
Source: Genes (Basel). 2024 Jun 14;15(6):787. doi: 10.3390/genes15060787 (PMC11203017; doi:10.3390/genes15060787)

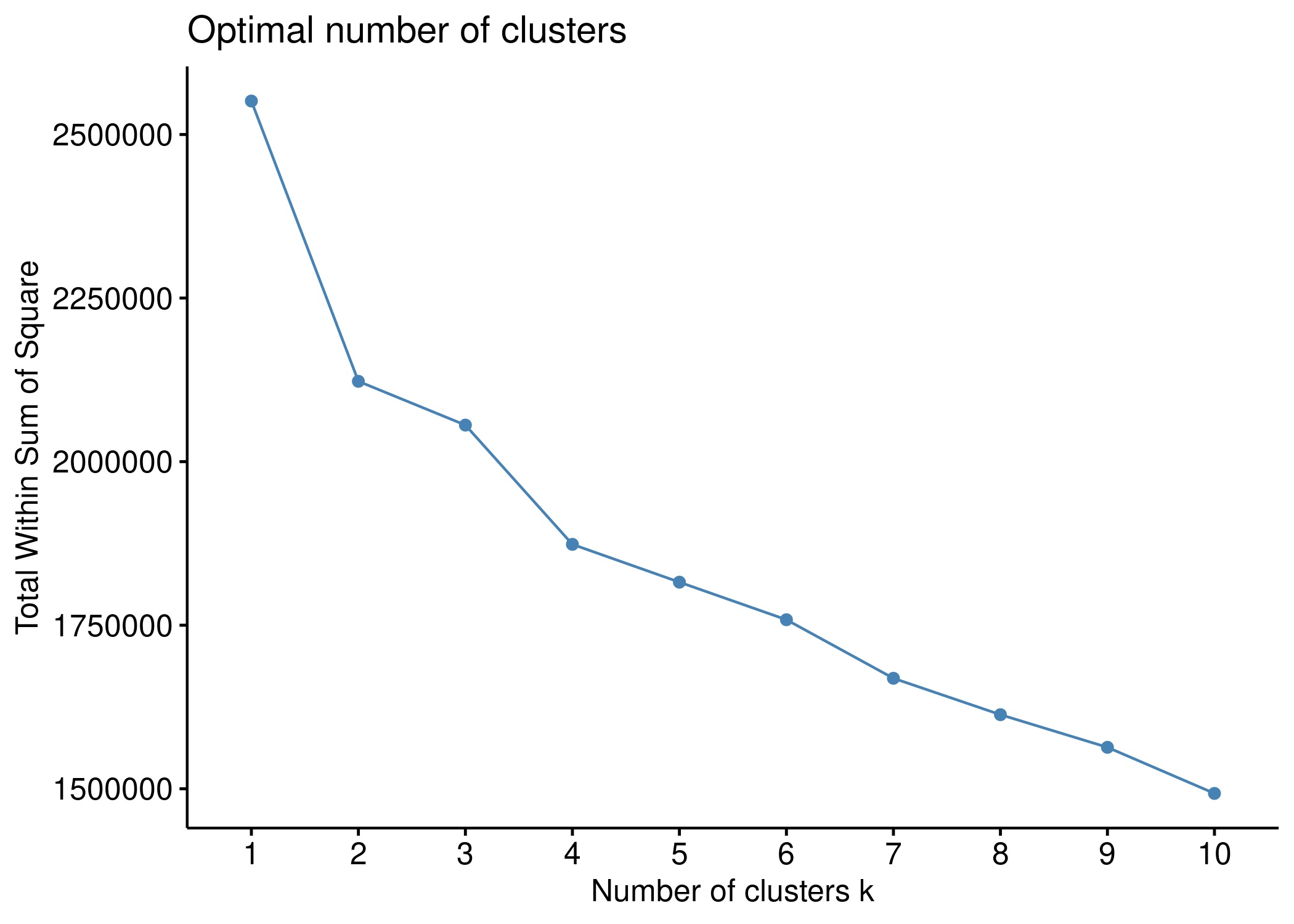

Supplement: Supplementary file 1 [file genes-15-00787-s001.zip › supplementary/Supplement Figure S1.jpg]
